# Manipulating orbital angular momentum of light with tailored in-plane polarization states

**DOI:** 10.1038/srep41001

**Published:** 2017-01-23

**Authors:** Luping Du, Zhongsheng Man, Yuquan Zhang, Changjun Min, Siwei Zhu, Xiaocong Yuan

**Affiliations:** 1 Nanophotonics Research Centre, Shenzhen University & Key Laboratory of Optoelectronic Devices and Systems of Ministry of Education and Guangdong Province, College of Optoelectronic Engineering, Shenzhen University, Shenzhen 518060, China; 2 School of Science, Shandong University of Technology, Zibo 255049, China; 3 Institute of Oncology, Tianjin Union Medicine Centre, Tianjin 300121, China

## Abstract

Generally, polarization and phase are considered as two relatively independent parameters of light, and show little interaction when a light propagates in a homogeneous and isotropic medium. Here, we reveal that orbital angular momentum (OAM) of an optical vortex beam can be modulated by specially-tailored locally linear polarization states of light under a tightly-focusing conditon. We perform both theoretical and experimental studies of this interaction between vortex phase and vector polarization, and find that an arbitrary topological charge value of OAM can be achieved in principle through vector polarization modulation, in contrast to the spin-orbital conversion that yields only the ± *ћ* OAM values through circular polarization. We verify the modulation of optical OAM state with vector beams by observing the orbital rotation of trapped particles.

Apart from linear momentum, a light beam can also carry angular momentum (AM) with orientation along the propagation direction. There are two categories of AMs: the spin angular momentum (SAM) that is associated with circular polarization, and the orbital angular momentum (OAM) arising from the helical wave front of a light beam. The SAM has two possible quantized values of ± *ћ* depending on the handedness of circular polarization^1^,^2^, where *ћ* is the Planck constant. While for a light beam with a spiral phase of exp(*ilφ*), it can carry an optical OAM of *lћ*^1^,^2^,^3^,^4^,^5^ where *l* is an integer known as the topological charge, indicating the repeating rate of 2π phase shifts azimuthally along the beam cross section. Such a vortex beam presents a phase singularity at the beam center, rendering a donut-shaped intensity profile^6^,^7^.

Since the original concept of optical OAM was pioneered by Allen *et al*. in 1992^3^, the OAM of light has excited a surge of academic interest because it brings a new degree of freedom of photons with unbounded quantum states. A great success has been achieved in the creation and manipulation of optical OAM and a varierty of practical applications were developed such as imaging and metrology^8^,^9^,^10^, atom optics^11^,^12^,^13^, nonlinear optics^14^,^15^,^16^, optical spanner^17^,^18^, quantum optics and information^19^,^20^,^21^,^22^, optical communications^23^,^24^,^25^,^26^,^27^,^28^,^29^,^30^, and others. The conventional ways to manipulate the optical OAM rely on the phase components/devices, e.g., spiral phase plates^31^,^32^,^33^,^34^, *q*-plates^35^, computer-generated holograms^36^,^37^, sub-wavelength gratings^38^ and plasmonic metasurfaces^39^,^40^,^41^. These devices can effectively control the outputting phase to generate beams with desired OAM modes. Generally, it is believed that polarization and phase are two relatively independent parameters of light that exhibt little interaction. Nevertheless, under specific conditions, the polarization of light was shown to enable the manipulation of optical OAM states via the spin-to-orbital AM conversion^42^. This is achieved by tightly-focusing a circularly polarized light (CPL) with a high numerical aperture (NA) lens, or by illuminating a CPL onto a plasminc vortex lens^43^. Such a kind of spin-orbital coupling allows for a faster and broadband manipulation of OAM states, in contrast to the aforementioned phase modulators that are generally wavelength dependent. However, due to the limited values of SAM (±*ћ*) per photon for CPL, it is still a challenge to access arbitrary value of optical OAM states through polarization.

Here, we predict in theory and validate in experiment a novel optical OAM manipultion process named “mode-splitting”, that enables accessing an arbitrary OAM state with vector polarization. Such a physical process arises under a high NA focusing configuration cooperated with an incident light with specially tailored locally linear state of polarization. Compared to the homogeneously-polarized light, a vector beam posesses a spatial-variant geometric configuration of polarizaiton states. A tightly-focusing system is able to change the vector properties of light. By tightly-focusing a vector-vortex beam, the OAM states of incident light can partly split into two OAM modes along the radial direction determined by the polarization states, giving rise to two categories of helical wavefront for the longitudinal electric field component. The carefully tailored locally linear state of polarization works like a special modulator that controls the two desired modes of the split OAM states and thus achieves manipulation of the optical OAM with arbitrary topological charges.

The mode-splitting of optical OAM states here is defined as the physical process by which the incident vector-vortex beam with a topological charge *l* can be separated into two different OAM states with topological charges of *l* + *m* − 1 and *l* − *m* + 1 along the radial direction, where *m* represents the azimuthal index of the polarization state. Figure 1a illustrates the concept of mode-splitting associated with optical OAM states. To realize such a physical process, we must have two elements: a high NA objective lens and a vector-vortex beam with specially-tailored locally linear state of polarization. State of polarization is one of the most salient features of light, which can be described with a Poincaré sphere (PS)^44^ as shown in Fig. 1b. Three variables *s*_1_, *s*_2_ and *s*_3_ denote the Stokes parameters of a point on the PS in the Cartesian coordinate system respectively, satisfying 
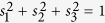
, and 2*θ* and 2*ϕ* stand for the latitude and longitude angles of this point in the spherical coordinate system. Mathematically, the state of polarization of any given polarized light can be described by the combination of a pair of orthogonal base vectors. Any two antipodal points on the PS are orthogonal and thus can be served as a pair of base vectors. When referring to a light beam with a locally linear polarization states, its state of polarization can be written by the unit vector as^45^,^46^


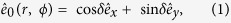


where *r* and *ϕ* are the polar radius and the azimuthal angle in the polar coordinate system, *δ* is a function determining the relative polarization distributions, 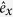 and 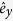 are the unite vectors along the *x* and *y* axis, respectively. We can find from Eq. (1) that the local state of polarization at any location is linearly-polarized, corresponding to the point at the equator on the PS. Such a kind of specially polarized light beam can be generated in the experiment with an interferometric arrangement^47^,^48^.

An example of a light beam carrying optical OAM is the Laguerre-Gaussian (LG_*l, p*_) laser modes^2^, where *l* and *p* are the numbers of interwined helices known as the topological charge and the additional concentric rings, respectively. Therefore, for a monochromatic paraxial locally linearly polarized light carrying a well defined value of OAM, its electric field can be written as


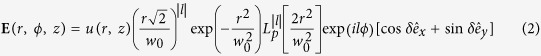


Here, *u(r, z*) is the radial profile of the field at position *z*, 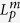 is the generalized Laguerre polynomials and *w*_0_ is the radius of the beam waist.

Tightly-focusing a vector-vortex beam is an excellent tool for detailed studies in nano-optics, e.g., particle acceleration^49^, microscopy^50^ and optical trapping^51^. The field distribution under the tightly-focusing condition can be derived by the vectorial diffraction theory built by Richards and Wolf 52. Such an analytical method was verified to agree well with the experimental results^53^,^54^, and it was employed to study the properties of light beams with peculiar polarization distributions^55^,^56^,^57^,^58^. Adopting the Richards-Wolf theory, we here calculate the tightly-focused electric field of vector-vortex beams described by Eq. (2), and each field component near the focus can be derived as (see Method for the details of derivation):


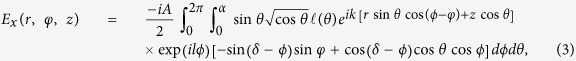



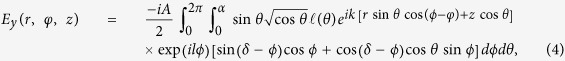



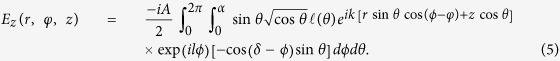


Here *A* is a constant, *k* is the wave-vector, *α* = sin^−1^(NA/*n*) is the maximum allowed incident angle determined by the NA of the objective lens where *n* is the refractive index of the medium in the image space, *ϕ* and *θ* are the azimuthal and polar angle, respectively.

To study the manipulation of the optical OAM based on the mode-splitting concept, we employ a vector-vortex beam with optical state of polarization given by


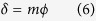


where *m* is the azimuthal index of polarization. For the sake of comparison, Figure 1c gives polarization and intensity distributions of three polarized beams with *m* = 1, 0 and −1, respectively. Different from the other two beams, it is homogeneously-polarized along the *x* axis when *m* = 0. While for *m* = ±1, the states of polarization are azimuthally-variant, hence presenting polarization singularity at the beam center. To further study the polarization properties, Fig. 1d gives respectively the local weights of the radial and azimuthal polarization components at different azimuthal angles in the beam cross section. Being distinct from the other two beams, the electric field is totally *p*-polarized in all directions for *m* = 1. While for the other two beams, the radial and azimuthal polarization components alternate along the azimuthal direction, with the alternating period equal to 2|*m* − 1|. It should be emphasized that the light vibration in the beam cross section for all these beams are linearly polarized, thus carrying no SAM.

For the given state of polarization described by Eq. (6), the *z*-component of the electric field under tight-focusing condition is (see Method for the details of derivation)


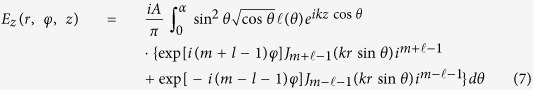


As can be seen from Eq. (7), there exhibit two modes of optical OAM in terms of (*m* + *l* − 1) *ћ* and −(*m* − *l* − 1) *ћ*. These two modes are separated from each other due to the modulation of the Bessel function of the first kind with different orders of (*m* + *l* − 1) and −(*m* − *l* − 1), indicating that the incident optical OAM state of *lћ* is split into the above two OAM modes. A special case must be emphasized that these two modes of OAM are equal to each other in the case of *m* = 1 (the well-known radially polarized beam with polarization distributions shown in Fig. 1c), indicating that there is no splitting.

The simulated electric field distributions in the focal plane under the illumination with the above three kinds of vector-vortex beams are shown in Fig. 2a–c. We consider *p* = 0 in all our configuration and simulations. Figure 2a shows the first case when *l* = 2 and *m* = 1, which is a radially polarized beam carrying optical OAM of 2*ћ*. The *z*-component electric field which dominates the total field has a donut-shaped intensity profile and exhibits two-fold helical phase distribution, implying that the incident radial polarization does not cause the split of optical OAM. However, when considering another vector-vortex beam with *l* = 2 and *m* = −1, which has the same topological charge as the first case but with a different polarization distribution. In such a case, there exhibit two modes of phase distribution [Fig. 2(d)]. One has no helical phase locating in the center due to the modulation of Bessel function J_0_(*x*); another one has four-fold helical phases appearing outside of the optics axis arising from the modulation of Bessel function J_−4_(*x*). These phase changes are manifested in the intensity profile of the *z*-polarized electric field component: a bright spot in the center and a quasi donut-shaped pattern around. When further increasing the charge to *l* = 4 while mantaining the incident polarization, one can see a two-fold helical phase inside and six-fold helical phase outside associated with the z-polarized component, as shown in Fig. 2c.

To experimentally verify the mode-splitting of optical OAM state predicted by our simulation, we measured the intensity distribution of the z-component electric field in the focal plane for the aforementioned vector-vortex beams (see Method for the details of the generation of the beams). The measurements were performed with a new method introduced in ref. 59, which employs a nanoparticle-on-film structure as a near-field probe that is sensitive to the out-of-plane field. The experimental results [Fig. 2d,e] are in excellent agreement with the calculated longitudinal field components [Fig. 2a–c], thus validating the realibility of our analytical model. Their cross-section comparisons along the *x*-axis for the three beams are plotted in Fig. 2g,h for a clear demonstration. More importantly, the experiment reveals the mode-splitting of optical OAM state controlled by the incident polarization under a high NA optical microscopy system.

To further confirm the feasibility of the above physical process, we carried out an optical trapping experiment with the tightly-focused electric field [see Methods for the details of the trapping experiment]. A linearly-polarized laser beam of 532-nm-wavelength and 150 mW power was transeferred respectiviely to the three vector-vortex beams (*l* = 2, *m* = 1; *l* = 2, *m* = −1 and *l* = 4, *m* = −1) to perform the optical trapping of neutral silica microspheres suspended in water. The silica spheres have a diameter of 0.70 μm. If there exhibts the mode-splitting of optical OAM state as predicted in Fig. 2, we should observe a difference in rotation between the incident beam modes of *l* = 2, *m* = 1and *l* = 2, *m* = −1, althouth they have the same incident optical OAM. In the meantime, the rotation between the beam modes of *l* = 2, *m* = 1and *l* = 4, *m* = −1 should be similar, althouth they have different incident optical OAM states.

Figure 3 shows the experimental results. For the first considered incident beam (*l* = 2, *m* = 1), as indicated with the sequentially-captured photographs (upper row), two trapped pariticles were rotated clockwise around the ring focus, with an orbital period of ∼1.13 s. When *m* was switched from positive (*m* = 1) to negative (*m* = −1), both of the particles were attracted into the center at the first and subsequently one of the particles moved away due to the Brownian motion, only the left one particle was trapped stably, as seen in the middle row. Further, when changing the topological charge of the vector-vortex beam from *l* = 2 to *l* = 4 while maintaining the azimuthal index of polaziation, another particle was attracted and rotated clockwise with the original one around the ring focus (bottom row), which is similar to the first case (upper row). The orbital period here is measured to be ∼1.81 s, which is slightly longer than that in the first case. This differences may arise from the different energy portion of the *z*-field component in the total field.

In summary, we reveal and demonstrate experimentally that the mode-splitting of optical OAM state occurs when a vector-vortex beam is manipulated under a tightly-focusing condition. The orbital angular momentum of the input light beam is separated into two different modes in the radial direction perpendicular to the optical axis controlled by the vector states of polarization, thus achieving the manipulation of optical OAM with arbitrary accessing topological charge values. These investigations may give rise to an extra degree of freedom on manipulating optical vortex beams.

## Methods

### Derivation for the focal field of tightly focused locally linearly polarized vortex beams

Based on the Richards-Wolf theory, we express the electric field near focus as a diffraction integral over the vector field amplitude **a**_1_ on a spherical aperture of focal radius *f*_1_.


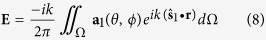


where the amplitude has the form:


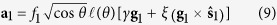


The unit vector **g**_1_ lies in the plane containing both the ray and the optical axis and is perpendicular to 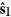, which is the propagation direction of the ray. *γ* and *ξ* are the decomposition coefficients in the radial and azimuthal directions, respectively, satisfying *γ*^2^ + *ξ*^2^ = 1. As the angle between the polarization direction and meridian plane remains unchanged after refraction through the lens, we get


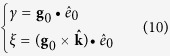


in which **g**_0_ represents the radial component in the object space, 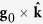 denotes the azimuthal component where 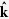 is a unit vector along the propagation direction, and the unit vector 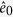 describes the polarization distributions of the incident field (Equation (1)). Let *θ* and *ϕ* represent the polar and azimuthal angles of the focused ray, the radial unit vector before and after refraction, can therefore be expressed as:


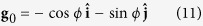



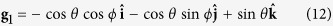


As a result,


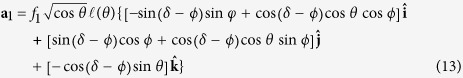


We employ the cylindrical coordinate system **r** = (*r, ϕ, z*) in the image space, with origin *r* = z = 0 located at the paraxial focus. Then, for point near the paraxial focus,


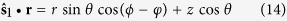


The Cartesian components of the electric field vector near focus then can be expressed as Eqs (3, 4, 5).

The integrations over *ϕ* can be accomplished using the identity:


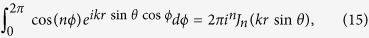


where *J*_*n*_(*kr* sin *θ*) is the Bessel function of the first kind of order *n*. For a given state of polarization described by Eq. (6), the *z*-polarized component of the electric field may then be expressed as Eq. (7).

### Generation and focusing of the vector-vortex beams

**Figure 4:**
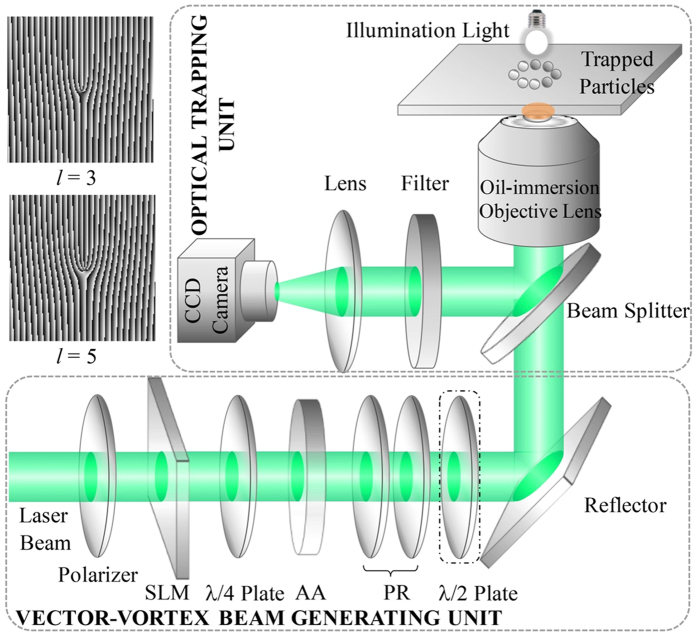
Experimental setup for generating the vector-vortex beams and characterizing their focusing properties. The insets give the computer generated holograms loaded onto the spatial light modulator (SLM) to generate the vector beams with topological charge of *l* = 3 and *l* = 5. AA: azimuthal analyzer.

The experiment setup for generating the vector-vortex beams and the corresponding optical trapping is presented in Fig. 4. The beam with *m* = 1 is achieved by passing a 532 nm linearly polarized beam sequentially through a spatial light modulator (loaded with desired computer generated hologram) and a quarter-wave plate. In a cylindrical coordinate system, the electrifc field for a right handed circularly polarized vortex beam can be expressed as:


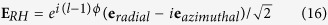


The desired vector-vortex beams can be achieved making use of an azimuthal analyzer for filtering out the radial components. Two half-wave plates are utilized subsequently for the radial/azimuthal polarization intercoversion. While for the beam with *m* = −1, it can be realized by simply adding another half-wave plate after the polarization rotator. The generated vector-vortex beams are subsequently tightly-focused by an oil-immersion objective lens [Olympus 100×, NA = 1.45] onto a glass substrate for trapping particles.

## Additional Information

**How to cite this article**: Du, L. *et al*. Manipulating orbital angular momentum of light with tailored in-plane polarization states. *Sci. Rep.* **7**, 41001; doi: 10.1038/srep41001 (2017).

**Publisher's note:** Springer Nature remains neutral with regard to jurisdictional claims in published maps and institutional affiliations.

## Figures and Tables

**Figure 1:**
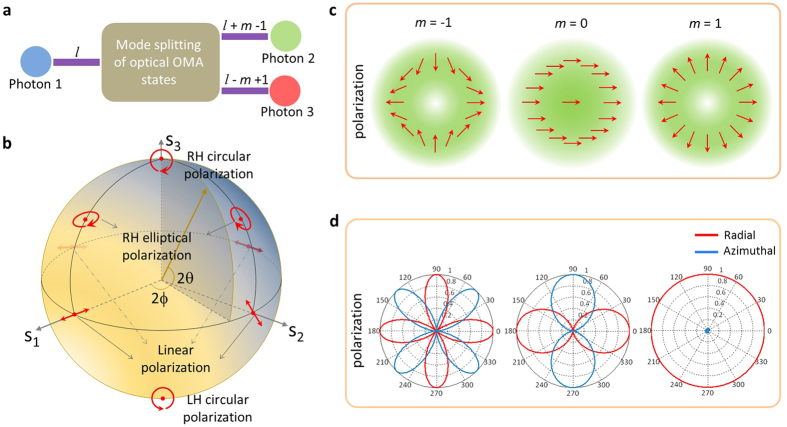
(**a**) The mode-splitting concept of optical OAM states, where *m* and *l* are the azimuthal index of polarization state and the topological charge of vortex phase, respectively. (**b**) Poincare sphere representation of polarization states for plane waves. The poles represent circular polarization, the equator linear polarization and the intermediate points elliptical polarization. The northern and southern hemispheres stand for the right-handed (RH) and left-handed (LH) elliptical polarization. The polarization states at antipodal points are orthogonal, and any other state of polarization is given as their linear combination. (**c)** Polarization distribution of three kinds of polarized beams in terms of *m* = −1, 0, and 1, respectively. (**d**) The weight of radial and azimuthal polarization component in the beam cross section of the three beams in (**c**).

**Figure 2:**
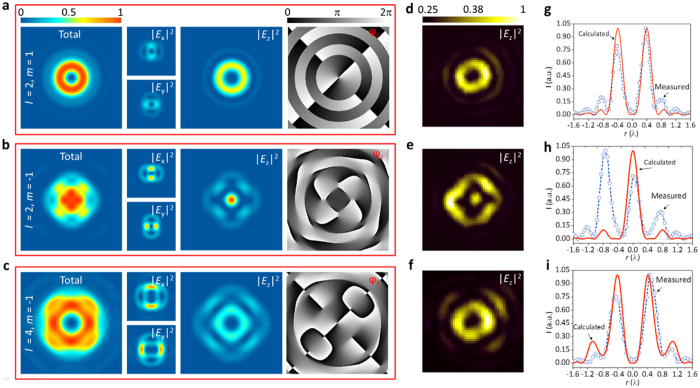
(**a–c**) Simulated intensity distributions of the total intensity and the x-, y- and z-polarized components in the focal plane of three vector-vortex beams in terms of *l* = 2 & *m* = 1, *l* = 2 & *m* = −1, and *l* = 4 & *m* = −1, where *m* and *l* are the azimuthal index of polarization state and vortex phase, respectively. The last column gives the phase patterns of the z-polarized components. (**d**–**f**) Experimentally measured intensity distributions of the z-polarized components by a near-field mapping technique. (**g**–**i)** Cross-section intensity comparison of the normalized measured and the calculated z-polarized components along the *x-*axis.

**Figure 3:**
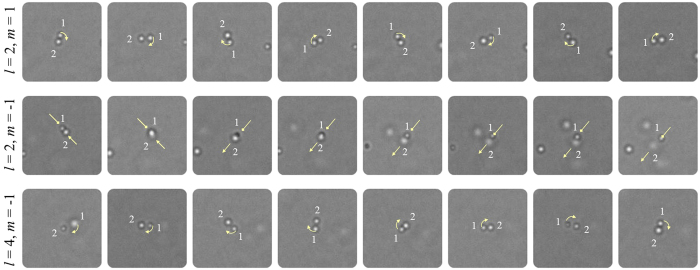
Snapshots of the motion of trapped particles around the focus under the illumination with the three aformentioned vector-vortex beams, respectively.
